# Maternal transmission of mitochondrial diseases

**DOI:** 10.1590/1678-4685-GMB-2019-0095

**Published:** 2020-03-02

**Authors:** Marcos R. Chiaratti, Carolina H. Macabelli, José Djaci Augusto, Mateus Priolo Grejo, Anand Kumar Pandey, Felipe Perecin, Maite del Collado

**Affiliations:** ^1^ Universidade Federal de São Carlos, Departamento de Genética e Evolução, Laboratório de Genética e Biotecnologia, São Carlos, SP, Brazil; ^2^ Lala Lajpat Rai University of Veterinary and Animal Sciences, Hisar, Haryana, India.; ^3^ Universidade de São Paulo, Faculdade de Zootecnia e Engenharia de Alimentos, Departamento de Medicina Veterinária, Laboratório de Morfofisiologia Molecular e Desenvolvimento, Pirassununga, SP, Brazil.

**Keywords:** Oocyte, germline, mitochondrial dynamics, mtDNA, metabolism

## Abstract

Given the major role of the mitochondrion in cellular homeostasis, dysfunctions of this organelle may lead to several common diseases in humans. Among these, maternal diseases linked to mitochondrial DNA (mtDNA) mutations are of special interest due to the unclear pattern of mitochondrial inheritance. Multiple copies of mtDNA are present in a cell, each encoding for 37 genes essential for mitochondrial function. In cases of mtDNA mutations, mitochondrial malfunctioning relies on mutation load, as mutant and wild-type molecules may co-exist within the cell. Since the mutation load associated with disease manifestation varies for different mutations and tissues, it is hard to predict the progeny phenotype based on mutation load in the progenitor. In addition, poorly understood mechanisms act in the female germline to prevent the accumulation of deleterious mtDNA in the following generations. In this review, we outline basic aspects of mitochondrial inheritance in mammals and how they may lead to maternally-inherited diseases. Furthermore, we discuss potential therapeutic strategies for these diseases, which may be used in the future to prevent their transmission.

## Introduction

The mitochondrion gained its deserved reputation in cell biology due to its role as the cellular powerhouse, with most of the adenosine triphosphate (ATP) in eukaryotic cells being supplied by this organelle (Wallace, 2013). However, mitochondria play several functions in the cell that far exceed the role in ATP generation. These are linked with buffering of Ca^+2^ levels, innate immunity, apoptosis and biogenesis of iron-sulfur clusters (Yasukawa *et al.*, 2009; Naon and Scorrano 2014; Stehling *et al.*, 2014). Moreover, mitochondria closely interact with other organelles such as the endoplasmic reticulum (ER) and regulate several pathways in the cell (de Brito and Scorrano, 2009; Betz *et al.*, 2013; Chen *et al.*, 2014; Carreras-Sureda *et al.*, 2017; Xu *et al.*, 2017). As a result, perturbations in mitochondrial function may dramatically disturb cellular homeostasis, resulting in several common diseases in humans (Bach *et al.*, 2003; Chen *et al.*, 2007, 2010; Schaefer *et al.*, 2008; Misko *et al.*, 2012; Schon *et al.*, 2012; Sebastian *et al.*, 2012; Eschbach *et al.*, 2013; Payne *et al.*, 2013; Schneeberger *et al.*, 2013; Pareyson *et al.*, 2015; Ramírez *et al.*, 2017).

Amongst mitochondria-associated diseases, those primarily linked to mitochondrial DNA (mtDNA) mutations have been a topic of great interest given their severe outcome and unclear pattern of inheritance (Craven *et al.*, 2017). However, mtDNA mutations can also associate with nuclear mutations, leading to common diseases in humans such as cancer, diabetes, Alzheimer, and Parkinson (Wallace 2011; Schon *et al.*, 2012; Stewart and Chinnery 2015). Thereby, recent findings have associated obesity with mitochondrial dysfunction in oocytes and increased risk of metabolic diseases in offspring (Wu *et al.*, 2015; Saben *et al.*, 2016). In mammals, mitochondria are uniparentally transmitted by females (Sutovsky *et al.*, 1999). Thus, maternal mitochondria are replicated during early embryogenesis to colonize somatic and germline tissues (St John, 2019). As a result, mitochondrial abnormalities present in oocytes can be perpetuated and lead to disease in offspring (Payne *et al.*, 2013; Saben *et al.*, 2016; Craven *et al.*, 2017; Wei *et al.*, 2019). In this review, we outline basic aspects of mitochondrial transmission in mammalian germline and how they may lead to maternally inherited diseases. Furthermore, we discuss potential therapeutic strategies for these diseases, which may be used in the future to prevent their transmission.

## Basic aspects of mitochondria

Mitochondria are double-membrane organelles with two distinct compartments, the inter-membrane space and the matrix. Most enzymes taking part in oxidative phosphorylation of energetic molecules (i.e., sugars, fats and proteins), including those of the Krebs cycle, are located in the mitochondrial matrix. The energy extracted from these molecules is then used by three (I, III and IV) out of four complexes imbedded in the inner mitochondrial membrane to pump H^+^ from the matrix to the inter-membrane space. This creates a difference in electric potential (the mitochondrial membrane potential – ΔΨ_m_). In turn, a fifth complex (V) phosphorylates ADP into ATP using the electrochemical energy derived from the H^+^ return to the matrix.

Mitochondria harbor their own genome, the mtDNA, which in mammals is ~16.5-kb long and encodes for 13 mRNAs, 2 rRNAs, and 22 tRNAs. These genes are essential for ATP synthesis in mitochondria as the 13 mtDNA-encoded proteins play key roles in complexes I, III, IV, and V of the electron transport chain. However, nearly 1,200 different proteins are present in mitochondria (i.e., complexes I to V are composed of ~80 proteins), most of which are encoded in the nucleus, translated in the cytoplasm and imported by mitochondria. Proteins regulating mtDNA replication, transcription and repair are similarly derived from the nucleus. Therefore, although mtDNA-encoded proteins are essential for ATP production in mitochondria, the nucleus exerts a broader role in regulating mitochondrial function (Garesse and Vallejo, 2001; Scarpulla, 2002; Battersby *et al.*, 2003).

Hundreds to thousands of mitochondria are present in each cell (Wassarman and Josefowicz, 1978; Jansen and De Boer, 1998; Motta *et al.*, 2000). These are, albeit, not isolated from each other. Actually, through repeated cycles of fusion and fission, mitochondria exchange membranes, solutes, metabolites, proteins, RNAs, and mtDNAs, resulting in electrically coupled organelles. The balance of fusion to fission also regulates mitochondrial number, morphology, transport, function, and turnover, which is collectively known as mitochondrial dynamics (Mishra and Chan, 2014). Both, fusion- and fission-deficient cells exhibit mitochondrial heterogeneity and dysfunction (Eura *et al.*, 2003; Chen *et al.*, 2003, 2005; Ishihara *et al.*, 2009; Udagawa *et al.*, 2014; Wakai *et al.*, 2014), supporting the importance of these events to mitochondrial health. In keeping with this, fragmentation of the mitochondrial network has been associated with a low bioenergetic state (i.e., in oocytes), while its elongation implies a high bioenergetic yielding, such as that of liver, muscle, and brain (Bach *et al.*, 2003; Zorzano *et al.*, 2015; Schrepfer and Scorrano 2016).

Several proteins regulate mitochondrial fission, with the Dynamin-related protein 1 (DRP1) being the best characterized (Ishihara *et al.*, 2009). DRP1 is a cytosolic protein that is recruited to mitochondria by multiple receptors, including mitochondrial fission factor (MMF), mitochondrial dynamic proteins of 49 kDa (MID49) and 51 kDa (MID51), and fission 1 (FIS1) (Mishra and Chan, 2014; Schrepfer and Scorrano 2016). In turn, the optic atrophy 1 (OPA1) regulates inner membrane fusion and cristae remodeling (Olichon *et al.*, 2002, 2003; Cipolat *et al.*, 2004; Griparic *et al.*, 2004; Pernas and Scorrano 2016), whereas mitofusins 1 (MFN1) and 2 (MFN2) regulate outer membrane fusion (Chen *et al.*, 2003, 2005, 2007, 2010; Ishihara *et al.*, 2004; Schrepfer and Scorrano, 2016). Mitochondrial fusion is initiated by homo and heterotypic interaction of MFN1 and MFN2 from two adjacent organelles (Ishihara *et al.*, 2004; Schrepfer and Scorrano, 2016). Given that MFN2 is present on the ER membrane, it also regulates ER-mitochondria tethering (de Brito and Scorrano, 2008). This connection, known as ER mitochondria-associated membranes (MAMs), has been shown to play an essential role in the regulation of ER, mitochondrial, and cellular functions (Ngoh *et al.*, 2012; Hamasaki *et al.*, 2013; Schneeberger *et al.*, 2013; Muñoz *et al.*, 2014; Carreras-Sureda *et al.*, 2017; Pathak and Trebak, 2018). MFN2 downregulation is associated with decreased expression of subunits of the Krebs cycle and electron transport chain, reduced oxygen consumption, lower ΔΨ_m_, and increased reactive oxygen species (ROS) (Santel and Fuller, 2001; Yasukawa *et al.*, 2009; Ngoh *et al.*, 2012; Wakai *et al.*, 2014; Filadi *et al.*, 2015; Schrepfer and Scorrano, 2016). These effects of MFN2 seem to be more evident in muscle, liver and hypothalamic neurons, tissues in which expression of MFN2 is enhanced (Chen *et al.*, 2007; Chen *et al.*, 2010; Schneeberger *et al.*, 2013; Schrepfer and Scorrano 2016). MFN2 expression has also been inversely linked with ER stress, insulin signaling and diabetes (Bach *et al.*, 2003; Mingrone *et al.*, 2005; Sebastian *et al.*, 2012; Schneeberger *et al.*, 2013; Zorzano *et al.*, 2015; Sarparanta *et al.*, 2017).[Bibr B98]

## Mitochondria in female germ cells

The earliest stages of embryogenesis are characterized by rapid cell division (i.e., cleavage) that gives rise to blastocysts. During these stages, the embryo relies on maternal factors inherited from the oocyte (i.e., mRNAs, proteins and mitochondria), as the embryonic genome is transcriptionally inactive. Also, in agreement with the “embryo silent” hypothesis, mitochondria show low activity during these stages to protect embryonic cells from oxidative damage (Leese, 2012). At the blastocyst stage, increased protein synthesis and blastocoel expansion is accompanied by upregulation of mitochondrial activity in cells that give rise to extraembryonic tissues (i.e., the trophectoderm) (Trimarchi *et al.*, 2000; May-Panloup *et al.*, 2005; Hashimoto *et al.*, 2017; St John, 2019). Activation of mitochondrial function is postponed, however, in the inner cell mass that originates the embryo proper. Mitochondrial architecture and function seem to remain underdeveloped in cells committed with germline specification, and mtDNA replication is only resumed with primordial germ cell (PGC) differentiation (Wassarman and Josefowicz, 1978; Motta *et al.*, 2000; Cree *et al.*, 2008; Wai *et al.*, 2008; St John *et al.*, 2010; Floros *et al.*, 2018; Chiaratti *et al.*, 2018; St John, 2019).

Among the hundreds of cells in the developing fetus, PGCs originate from a few dozen located at the basis of allantois. Yet, after migration to the genital ridge, PGCs proliferate quickly to generate in females millions of oogonia (Leitch *et al.*, 2013). After entering meiosis, these primary oocytes receive a cover layer of somatic pre-granulosa cells, giving rise to primordial follicles still during fetal life. These follicles constitute the ovarian reserve that females carry throughout their reproductive life (Oktem and Urman, 2010). After puberty, the ovary provides an adequate environment for follicle growth and maturation (Clarke, 2017). During this period, the oocyte stockpiles several molecules that are required later during embryogenesis. This includes a ~1,000-fold increase in mitochondria (Jansen and De Boer, 1998; Cree *et al.*, 2008; Wai *et al.*, 2008; St John, 2019), which accounts for the largest mitochondrial content amongst all cells in mammals. In spite of this, mitochondria display several characteristics that suggest they are immature and low functional in oocytes (Arhin *et al.*, 2018). In fact, oocytes lacking the pyruvate dehydrogenase E1 alpha 1 (PDHA1), a key gene required for mitochondrial activity, successfully develop during most part of oogenesis and are ovulated (Johnson *et al.*, 2007). Thus, although mitochondria do play an essential role during the final steps of oocyte development, the “embryo silent” hypothesis likely extends to oogenesis too (Arhin *et al.*, 2018). Accordingly, somatic cells surrounding the oocyte (i.e., cumulus cells) provide the oocyte with several energetic molecules, including amino acids, cholesterol, pyruvate, AMP, and ATP (Su *et al.*, 2007, 2009; Sugiura *et al.*, 2007). Moreover, the adenosine salvage pathway seems to be a key source of ATP, giving it can be generated from abundant amounts of cyclic AMP (cAMP) present in oocytes (Scantland *et al.*, 2014).

If mitochondria are not highly active in oocytes, why are they present in massive amounts before fertilization? This can be, at least, partially explained by downregulation of mitochondrial biogenesis during early embryogenesis; mitochondria are segregated among hundreds of embryonic cells without any increase in number up to the time of embryo implantation (Pikó and Taylor, 1987; Thundathil *et al.*, 2005; Cree *et al.*, 2008; Wai *et al.*, 2008; St John, 2019). Therefore, a threshold number of mitochondria is necessary in oocytes to assure that every embryonic cell will inherit a minimum complement of mitochondria (Chiaratti and Meirelles, 2010; Wai *et al.*, 2010). In keeping with this idea, extensive fragmentation of the mitochondrial network in oocytes allows for efficient segregation of mitochondria during early embryogenesis (Ashley *et al.*, 1989; Cree *et al.*, 2008; Ferreira *et al.*, 2010; Lee *et al.*, 2012b). Upregulation of pro-fission proteins (i.e., DRP1) and downregulation of MFN2 likely supports mitochondrial fragmentation during oogenesis (Udagawa *et al.*, 2014; Machado *et al.*, 2018; Hou *et al.*, 2019; Zhang *et al.*, 2019b). However, oocytes do retain fusion competence, as loss of DRP1 leads to mitochondrial elongation (Udagawa *et al.*, 2014). Moreover, MFN1 is required for oocyte growth and ovulation; MFN1 loss impairs oocyte-somatic cell communication, disrupting folliculogenesis (Machado *et al.*, 2018; Hou *et al.*, 2019; Zhang *et al.*, 2019a,b).

## Mitochondrial diseases originated from mtDNA mutations

Diseases caused by mutations in mtDNA are mostly severe and affect ~1 in 4,300 people all over the world (Schaefer *et al.*, 2008). In addition, almost every person (including healthy people) carries very low levels of mutant mtDNA (Payne *et al.*, 2013) that may be passed down to following generations and associate with late-onset diseases, such as Parkinson disease, Alzheimer disease, and common cancers (Poulton *et al.*, 2010;Wallace, 2011; Schon *et al.*, 2012; Gorman *et al.*, 2015; Stewart and Chinnery, 2015). With rare exceptions (Luo *et al.*, 2018), mitochondria are inherited exclusively from the mother (Wallace and Chalkia, 2013). This uniparental pattern of inheritance is explained by the presence of several thousand mitochondria in the ovulated oocyte, against only dozens in the sperm (Wai *et al.*, 2010). Additionally, the early embryo actively eliminates paternal mitochondria introduced into the oocyte during fertilization (Sutovsky *et al.*, 1999; Rojansky *et al.*, 2016). Although it is not clear why sperm mitochondria are excluded from the developing embryo, their elimination is in agreement with the “embryo silent” hypothesis, as sperm mitochondria are elongated, contain well-developed cristae and are highly active (Sutovsky *et al.*, 1999; Ford, 2004; Ruiz-Pesini *et al.*, 2007; Wai *et al.*, 2010; Rojansky *et al.*, 2016).

Mutations in mtDNA are much more frequent than in the nuclear DNA (Johnson and Johnson, 2001), which was initially thought to be explained by mtDNA proximity to ROS generation sites; the mitochondrial genome is attached to the inner mitochondrial membrane, close to complexes involved with the electron transport chain (Wallace, 2005). However, there is now data supporting that most mtDNA mutations originate from replication errors of the mtDNA polymerase (Kauppila *et al.*, 2017). In humans, mice, and flies, for instance, transition mutations, which are indicative of replication errors, are more common than transversions, which often result from oxidative damage (Tomas, 1993; Zheng *et al.*, 2006; Kennedy *et al.*, 2013; Itsara *et al.*, 2014). In fact, the machinery of DNA repair in the mitochondrion does not seem to be as effective as in the nucleus (Vermulst *et al.*, 2008; Maynard *et al.*, 2009; Kazak *et al.*, 2012; Muftuoglu *et al.*, 2014). Thus, intense replication of mtDNA during oogenesis makes it prone to replication errors (Wai *et al.*, 2008; Mahrous *et al.*, 2012; Wei *et al.*, 2019).

The existence of a DNA repair machinery inside mitochondria is well established, but not fully characterized (Scheibye-Knudsen *et al.*, 2015). Most genes encoding for factors involved in this machinery are shared with the nucleus; alternative variants of these genes allow for the protein to be targeted either to the nucleus or the mitochondrion (Muftuoglu *et al.*, 2014; Scheibye-Knudsen *et al.*, 2015). The best-known pathway of DNA repair in mitochondria is base excision repair (BER). Yet, several other enzymes involved with mismatch repair (MMR), non-homologous end joining (NHEJ), and direct repair have been reported in mitochondria (Maynard *et al.*, 2009, 2010; Ruhanen *et al.*, 2010; Halsne *et al.*, 2012; Kazak *et al.*, 2012; Sharma *et al.*, 2014; Scheibye-Knudsen *et al.*, 2015). Moreover, although homologous recombination (HR) has not been proved to contribute with mtDNA repair (Kazak *et al.*, 2012; Hagström *et al.*, 2014; Scheibye-Knudsen *et al.*, 2015), mitochondria do import RAD51, one of the most prominent enzymes of HR (Sage *et al.*, 2010; Chen, 2013). RAD51 has also been linked with mtDNA synthesis under replicative stress (Sage and Knight, 2013), and in oocytes RAD51 is required for mitochondrial function (Kim *et al.*, 2016).

**Figure 1:**
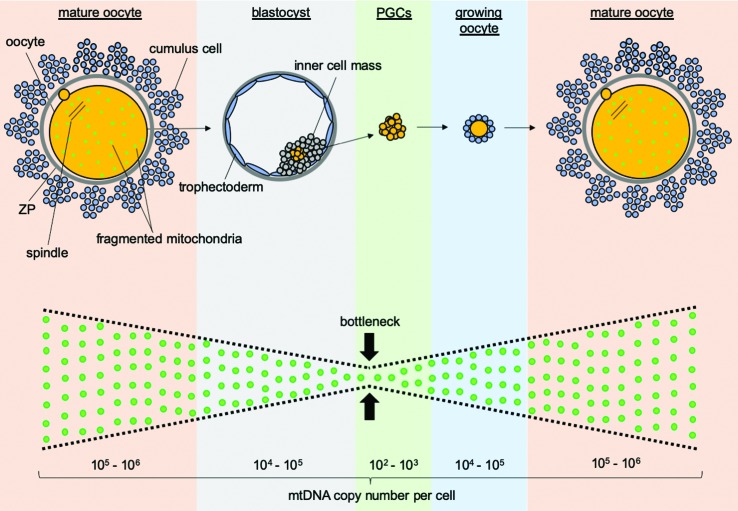
Mitochondrial kinetics in the female germline. Throughout germline development, the number of mitochondrial DNA (mtDNA) molecules per cell varies from 10^5^ - 10^6^ in mature oocytes (before fertilization), 10^2^ - 10^3^ in primordial germ cells (PGCs) and 10^5^ - 10^6^ back to mature oocytes. This variation in copy number accounts for the mitochondrial genetic bottleneck, which forces segregation of mtDNA molecules. In line with this, the mitochondrial network is fragmented in oocytes, allowing efficient partitioning of mitochondria among hundreds of cells until embryonic implantation. In addition, only few cells in the embryo differentiate into PGCs, supporting a sampling effect towards selection of a single mtDNA genotype to populate the future oocyte.

Given that most cells contain several mtDNA molecules, a *de novo* mutation creates a condition termed heteroplasmy, characterized by the co-existence of two or more mtDNA genotypes (i.e., wild-type and mutant mtDNAs) within the same cell or organelle. Heteroplasmy commonly protects the cell, as most mtDNA mutations are recessive. Unless the mutation level exceeds a critical threshold necessary to cause a biochemical defect (i.e., above 60-90%), the mutation effect will be masked by wild-type molecules (Schon *et al.*, 2012; Aanen *et al.*, 2014; Haig, 2016). In addition, a mechanism known as the mitochondrial genetic bottleneck (Hauswirth and Laipis, 1982; Olivo *et al.*, 1983; Hauswirth *et al.*, 1984; Jenuth *et al.*, 1996; Burgstaller *et al.*, 2018) acts in the germline to rapidly re-establish homoplasmy (i.e., the presence of a single mtDNA genotype). This mechanism is based on the absence of mtDNA replication during early embryogenesis, which forces wild-type and mutant mtDNAs to segregate. Also, few cells among the hundreds present in the embryo differentiate into PGCs, resulting in a sampling effect that efficiently selects one mtDNA genotype to populate the following generation (Stewart and Chinnery, 2015; Burgstaller *et al.*, 2018). However, the selected genotype can be either wild-type or mutant, generating genetic variability to be put to test at the cellular, organismal, or population level (Figure 1).

**Figure 2:**
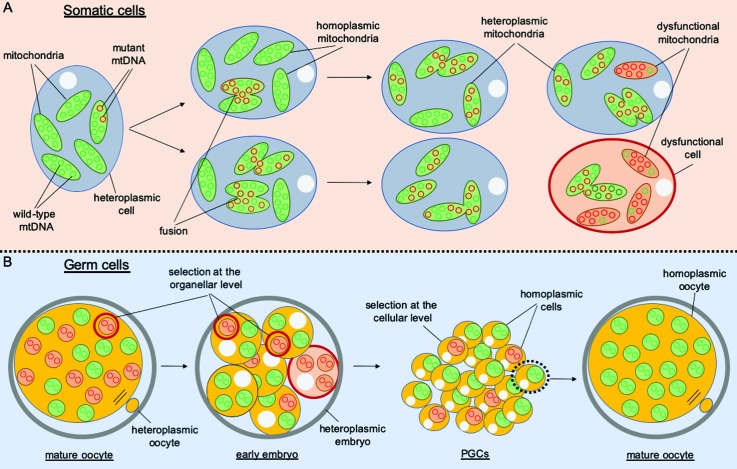
Mitochondrial DNA inheritance in somatic and germ cells. Different mitochondria in a single somatic cell (A) are interconnected by constant events of fusion and fission, allowing them to share membranes, solutes, metabolites, proteins, RNAs and DNA (mitochondrial DNA – mtDNA). Hence, when a mutation in mtDNA arises, it can rapidly spread throughout the mitochondrial network. In this case, mutant (red circles) and wild-type (green circles) mtDNAs may co-exist, which is known as heteroplasmy. In comparison, homoplasmic mitochondria contain a single mtDNA genotype, either mutant or wild-type. Unless the mutation level exceeds a critical threshold necessary to cause a biochemical defect (i.e., above 60-90%; red mitochondria), the mutation effect will be masked by wild-type molecules (green mitochondria with both mutant and wild-type mtDNA). In germ cells (B), downregulation of fusion likely minimizes heteroplasmy within mitochondria, enhancing selection at the organellar level (i.e, stronger association between mitochondrial genotype and phenotype). In addition, decreased fusion leads to mitochondrial fragmentation, enhancing mtDNA segregation among embryonic cells. Hence, decreased levels of mtDNA in primordial germ cells (PGCs) makes possible selection at the cellular level (i.e., stronger association between mitochondrial genotype and cellular phenotype). Thus, as a result of selection against deleterious mutations, mature oocyte from the next generation may contain lower levels of mutant mtDNA.

Mutations in mtDNA may vary considering their effect on mitochondrial function from neutral to deleterious. Among deleterious mutations, those affecting tRNA are the most frequent in humans. This is counter-intuitive though, as tRNA genes account for only 10% of the total coding capacity of mtDNA (Schon *et al.*, 2012). However, in comparison with protein-coding genes, tRNA mutations are considered to be less severe, as higher levels (above 90%) are required to cause a biochemical defect (Yoneda *et al.*, 1995). This finding is in agreement with several works that have provided evidence in support of purifying selection acting in germ cells against deleterious mtDNA mutations (Rand, 2008) (Figure 2). For instance, Stewart and colleagues have shown that mice with a burden of mtDNA mutations are less likely to transmit to offspring non-synonymous changes in protein-coding genes (Stewart *et al.*, 2008). In contrast, synonymous substitutions in protein-coding genes and mutations in tRNAs and rRNAs were present at higher levels (Stewart *et al.*, 2008). Similar observations have been reported for flies, mice, and humans (Sato *et al.*, 2007; Fan *et al.*, 2008; Freyer *et al.*, 2012; Sharpley *et al.*, 2012; Hill *et al.*, 2014; Ma *et al.*, 2014; Li *et al.*, 2016; Floros *et al.*, 2018; Wei *et al.*, 2019), suggesting a conserved mechanism of purifying selection was established early during evolution. Accordingly, Lieber *et al.* (2019) recently reported that mitochondrial fragmentation is required to drive selective removal of deleterious mtDNA during early oogenesis in *Drosophila*. Fragmentation likely enhances association between mitochondrial genotype and phenotype, favoring one genotype over another (Aanen *et al.*, 2014; Haig, 2016). Nonetheless, at least in *Drosophila*, this mechanism does not rely on autophagic elimination of mutant mtDNA. Instead, mitophagic proteins enable preferential replication of wild-type mtDNA to outcompete their mutant counterparts (Hill *et al.*, 2014; Ma *et al.*, 2014; Lieber *et al.*, 2019).

In spite of the mounting evidence in support of a filter against mutant mtDNA in the female germline, this is not a resolved issue. Actually, there are conflicting data arguing against this filter, which has been generating much debate over the topic (Burr *et al.*, 2018). Other questions involving the issue are: i) why would purifying selection be restricted to germline? ii) can one manipulate selection to avoid the accumulation of mutant mtDNA in somatic tissues? Whilst these questions remain unresolved, it is very likely that the purifying selection behaves differently for different mtDNA mutations and different nuclear genetic backgrounds.

## Transmission of metabolic diseases linked to mitochondria dysfunction

Obesity and type II diabetes are currently recognized as the most endemic diseases in the human population. The frequency of these syndromes is increasing over the years; currently, nearly half of worldwide population suffers from obesity (Barnett, 2019; Blüher, 2019). Obesity and type II diabetes share similar metabolic alterations and are believed to be highly correlated (Volaco *et al.*, 2018). Transmission of these diseases to the following generations can occur through both parents, yet the maternal contribution has been shown to be larger (Shankar *et al.*, 2008; Jungheim *et al.*, 2010; Rattanatray *et al.*, 2010; Ruager-Martin *et al.*, 2010; Luzzo *et al.*, 2012). In humans, for instance, offspring body mass index (BMI) correlated through three generations with maternal but not paternal BMI (Murrin *et al.*, 2012). Likewise, maternal overnutrition in mice leads to offspring that are glucose intolerant and present increased cholesterol and body fat (Jungheim *et al.*, 2010). These alterations can last up to the third generation, even when pups are fed a regular diet (Saben *et al.*, 2016). Although epigenetic alterations in the nucleus play a major role in the regulation of these effects (Agarwal *et al.*, 2018; Wang *et al.*, 2018), other maternal factors have also been taken into account (Wu *et al.*, 2015; Saben *et al.*, 2016).

Among the factors that contribute with maternal transmission of metabolic diseases, mitochondria are a main candidate giving their maternal-exclusive inheritance. In fact, mitochondrial defects in somatic tissues have been associated with obesity, diabetes and cardiovascular disease (Silva *et al.*, 2000; Sarparanta *et al.*, 2017; Ferey *et al.*, 2019). For instance, mtDNA mutations impacting mitochondrial function and ATP production link with abnormal insulin release and β-cell development, insulin resistance, and diabetes (Poulton *et al.*, 1998; Silva *et al.*, 2000; Kaufman *et al.*, 2015). In this context, Tanaka *et al.* (2002) demonstrated that single nucleotide polymorphisms in mtDNA (mtSNPs) may result in decreased energy expenditure, leading to obesity. Moreover, several studies have associated mtSNPs with type II diabetes and obesity (Rivera *et al.*, 1999; Fuku *et al.*, 2002; Okura *et al.*, 2003; Guo *et al.*, 2005). These mtSNPs can be located in genes coding for rRNAs, tRNAs, mRNAs (i.e., *MT-CYB* or *MT-ATP6*), and even in the non-coding region of mtDNA, the D-loop. Similarly, it was recently described that several mtDNA mutations in tRNAs lead to polycystic ovarian syndrome and metabolic alterations (Ding *et al.*, 2018), both closely related to type II diabetes and obesity. Altogether, these findings provide evidence that mtDNA mutations may underpin maternal transmission of metabolic diseases.

Apart from mtDNA mutations, mitochondrial damage in oocytes has also been linked with increased risk of metabolic diseases in offspring. Obesity leads to increased lipid content in the follicular fluid, cumulus cells, and oocytes, which in turn damage organelles such as mitochondria and the ER (Wang *et al.*, 2009; Wu *et al.*, 2010; Fullston *et al.*, 2015; Ruebel *et al.*, 2017). Impaired ER function can lead to activation of the unfolded protein response (UPR) and Ca^+2^ release, further disrupting mitochondrial function (i.e., decreased Δψm and increased ROS) and oocyte homeostasis (Wu *et al.*, 2010, 2015; Luzzo *et al.*, 2012; Hou *et al.*, 2016). Besides impacting oocyte competence and fertility (Wu *et al.*, 2015; Pasquariello *et al.*, 2019), these mitochondrial abnormalities can be passed down to the following generations, increasing their risk to develop metabolic diseases (Saben *et al.*, 2016). Hence, mice born to pregnant females under a high-fat/high-sucrose diet have impaired peripheral insulin signaling which associates with abnormal mitochondrial function and dynamics in skeletal muscle up to the third generation (Saben *et al.*, 2016). Similar mitochondrial abnormalities were present in oocytes from the first and second generations, even though these were fed a regular diet (Saben *et al.*, 2016). Therefore, apart from epigenetic alterations in the nucleus, mitochondria also contribute with the metabolic programing resulting from maternal overnutrition. Given that epigenetic marks in mtDNA regulate expression of this genome (Kobayashi *et al.*, 2012; Sun *et al.*, 2013; Sirard, 2019), it remains to be investigated whether these can also explain maternal transmission of metabolic diseases.

## Treatment options for preventing mitochondrial disease transmission

Due to the poor understanding of the mechanisms regulating transmission of mitochondria-related diseases, there are few treatment options available to prevent their inheritance to the following generations (Craven *et al.*, 2017). With respect to non-genetic alterations in mitochondria, the oocyte might benefit from treatments performed before fertilization, during the *in vitro* maturation. The idea is to expose the oocyte for a period of ~24 h to drugs such as L-carnitine, rosiglitazone, salubrinal, or BGP-15, which potentially enhance mitochondria activity, decrease lipid content, and mitigate ER stress. In fact, treatments involving one or more of these drugs have been shown to mitigate the defects in the oocyte and the next generation (Wu *et al.*, 2010, 2015; Dunning and Robker, 2012; Liang *et al.*, 2017). However, a major challenge in making these treatments available is to overcome the side effects of *in vitro* maturation (Lonergan and Fair, 2016; Yang and Chian, 2017). Given this is a critical period of oocyte development, which encompasses meiotic resumption from prophase I (dictyate) to metaphase II, any perturbation in oocyte homeostasis may lead to mis-segregation of chromosomes and aneuploidy (Greaney *et al.*, 2017; Danadova *et al.*, 2017). In addition, *in vitro* maturation on its own leads to metabolic alterations that mimic those of oocytes from obese donors (i.e., mitochondrial dysfunction and increased lipid content), potentially impacting the next generation (Farin *et al.*, 2006; Li *et al.*, 2014; del Collado *et al.*, 2017a; del Collado *et al.*, 2017b; Wang *et al.*, 2018). Thus, these alternatives are not currently available in humans.

**Figure 3:**
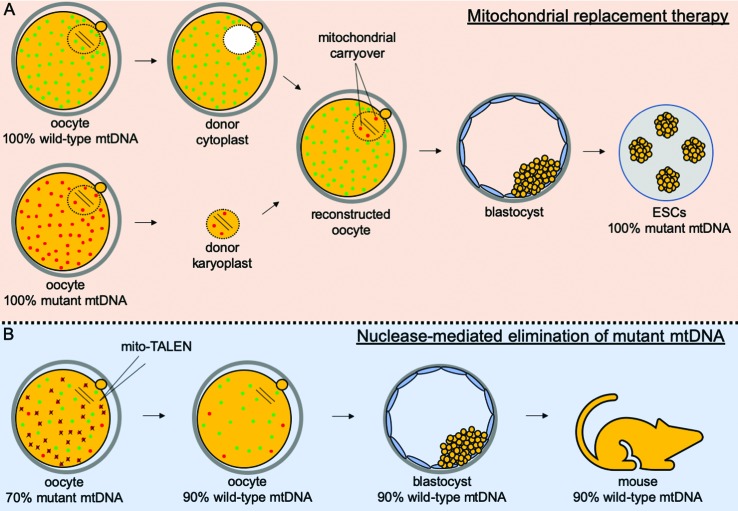
New technologies for preventing inheritance of mitochondrial diseases. The mitochondrial replacement therapy (MRT; A) proposes the replacement of a patient’s mitochondria in oocytes by donor mitochondria. Towards that, mature oocytes arrested at the metaphase-II stage are collected from the patient and a donor. While the patient’s oocytes are supposed to contain mutant (red) mitochondrial DNA (mtDNA), donor oocytes should contain only wild-type (green) mtDNA. Next, the spindle is removed from the patient’s oocyte (donor karyoplast) to be injected into the donor oocyte from which the spindle was previously removed (donor cytoplast). Fertilization of the reconstructed oocyte should lead to a blastocyst, which can be used for embryonic stem cell (ESC) derivation. Although MRT allows transplantation of karyoplast with minimal carryover (~1%) of mutant mtDNA, recent data have provided evidence of a reversal in ESCs back to 100% mutant mtDNA (Hyslop *et al.*, 2016; Kang *et al.*, 2016). An alternative strategy to MRT is the nuclease-mediated elimination of mutant mtDNA (B), which relies on the use of mitochondrial-targeted restriction endonucleases (mito-TALENs). These nucleases are designed to selectively cut mutant mtDNA, but not wild-type molecules. However, ~10% of targeted molecules were shown to be left uncut in newborns after use of mito-TALENs (Reddy *et al.*, 2015).

An alternative option to treat oocytes harboring mitochondria abnormalities, particularly those caused by mtDNA mutations, is known as mitochondrial replacement therapy (MRT; Figure 3). This method involves replacement of abnormal mitochondria in the oocyte by functional ones provided by a donated oocyte (Wolf *et al.*, 2017). More specifically, ovulated oocytes at the metaphase-II stage are collected from both the patient and a “healthy” donor not containing mitochondrial abnormalities. With the aid of a micromanipulation set, the spindle from the donated oocyte is replaced by the patient’s spindle. The resulting oocyte containing the patient’s spindle and donated mitochondria is then fertilized to allow development to term. Provided that the large majority of mitochondria is replaced by donated ones, MRT has virtually the potential to prevent transmission of mitochondrial diseases. Yet, ~1% of mitochondria from the patient’s oocyte are transferred along with the spindle. This level can be even higher (up to 4%) when pronuclear zygotes are used instead of metaphase-II oocytes, which can lead in ~15% of cases to a reversal back to the patient’s mtDNA (Hyslop *et al.*, 2016; Kang *et al.*, 2016). Although hard to explain, rapid mtDNA segregation and bottleneck during preimplantation development might account for these quick shifts in mtDNA genotype (Lee *et al.*, 2012a; Freyer *et al.*, 2012). Alternatively, it has been proposed that a specific population of mtDNA is tagged in oocytes (i.e., from spindle-surrounding mitochondria) for replication during early development (Wolf *et al.*, 2017). No matter the mechanism underlying these unexpected results, they highlight the need for careful studies before the clinical practice of MRT (Wolf *et al.*, 2017; Craven *et al.*, 2018).

With the advances in genome editing technologies, another potential strategy to prevent transmission of mitochondrial abnormalities is the targeted elimination of mutant mtDNA in oocytes or early embryos (Figure 3). As a proof of concept, Reddy *et al.* (2015) used mitochondrial-targeted restriction endonucleases (mito-TALENs) to selectively eliminate mutant mtDNA in mice and humans. Although this strategy proved efficient, ~10% of targeted molecules (i.e., mutant mtDNA) were left in oocytes, embryos and offspring produced after the use of mito-TALENs. Moreover, given that the mtDNA is not replicated during early embryogenesis (Pikó and Taylor, 1987; Thundathil *et al.*, 2005; Cree *et al.*, 2008), the use of mito-TALENs resulted in mtDNA-depleted embryos (Reddy *et al.*, 2015). Although in the newborns the content of mtDNA was normal (Reddy *et al.*, 2015), the lower levels of mtDNA (and likely of mitochondria too) in oocytes and embryos could lead to poorer developmental rates (Wai *et al.*, 2010). Based on these uncertainties, mito-TALENs are not currently taken as a viable alternative to prevent transmission of mtDNA-linked diseases (Wolf *et al.*, 2017).

## Final considerations

Mitochondrial abnormalities have been linked with maternal transmission of important diseases in humans. Among these, mtDNA mutations in oocytes can be transmitted to the following generations and cause severe diseases. In addition, maternal obesity damages mitochondria in oocytes, leading to poor fertility and increased risk of metabolic diseases in offspring. Understanding how mitochondrial abnormalities are established and transmitted are of fundamental importance to mitigate their incidence in the human population. Moreover, treatment options involving manipulation of oocytes and early embryos are currently under consideration and may become available in the future to prevent transmission of mitochondria-associated diseases.
